# CRISPR‐PLANT v2: an online resource for highly specific guide RNA spacers based on improved off‐target analysis

**DOI:** 10.1111/pbi.13025

**Published:** 2018-12-13

**Authors:** Bastian Minkenberg, Jianwei Zhang, Kabin Xie, Yinong Yang

**Affiliations:** ^1^ Intercollege Graduate Degree Program in Plant Biology The Huck Institutes of the Life Sciences The Pennsylvania State University University Park State College PA USA; ^2^ Department of Plant Pathology and Environmental Microbiology The Pennsylvania State University University Park State College PA USA; ^3^ College of Plant Sciences and Technology National Key Laboratory of Crop Genetic Improvement Huazhong Agricultural University Wuhan China; ^4^ Arizona Genomics Institute University of Arizona Tucson AZ USA; ^5^ Present address: Innovative Genomics Institute University of California Berkeley CA USA

**Keywords:** Genome editing, CRISPR‐Cas9, guide RNA, off‐target prediction

Dear Editor,

Since the beginning of CRISPR/Cas9's use for genome editing, reports of off‐targets have caused concerns among users. Previous studies identified three main types of off‐targets. The first type of off‐targets are sequences at other 5’‐NGG‐3’ protospacer adjacent motifs (PAMs) that are identical or have a small number of mismatches or substitutions (Fu *et al*., 2013; Tsai *et al*., 2015). The second type of off‐targets are sequences at other 5’‐NGG‐3’ PAMs that have insertions or deletions compared to the gRNA spacer or the targeted DNA (Lin *et al*., 2014). The RNA or DNA will form a small bulge with the remaining nucleotides perfectly annealing to facilitate Cas9 activity. In some cases, the off‐target activity detected on these sites was higher than the on‐target activity (Lin *et al*., 2014). The last type of off‐targets is cleavage of sequences with the alternative 5’‐NAG‐3’ PAM (Fu *et al*., 2013; Tsai *et al*., 2015). Several bioinformatics tools have been developed to predict specific gRNA spacers that are different enough from other sites in the genome. However, these tools are not reliable and could not predict all off‐target sites found in unbiased genome‐wide studies such as GUIDE‐seq (Tsai *et al*., 2015). Therefore, predicting gRNA specificity and activity is more complex than previously thought, and it is necessary to evaluate and improve the current bioinformatics tools.

We found in our evaluation two main reasons for the inadequate performance of these tools. First, they were developed without consideration of all off‐target types, and second, they use aligners and options that are not suitable for small sequences like 20 nt long spacers. We developed a new strategy to predict off‐targets that outperforms all of the tested tools (Figure 1a,d). We used this strategy to create lists of highly specific gRNA spacers for seven genomes of model and crop plants. The results were used to update the CRISPR‐PLANT (Xie *et al*., 2014) website to version 2, which is now available at http://www.genome.arizona.edu/crispr2/.

**Figure 1:**
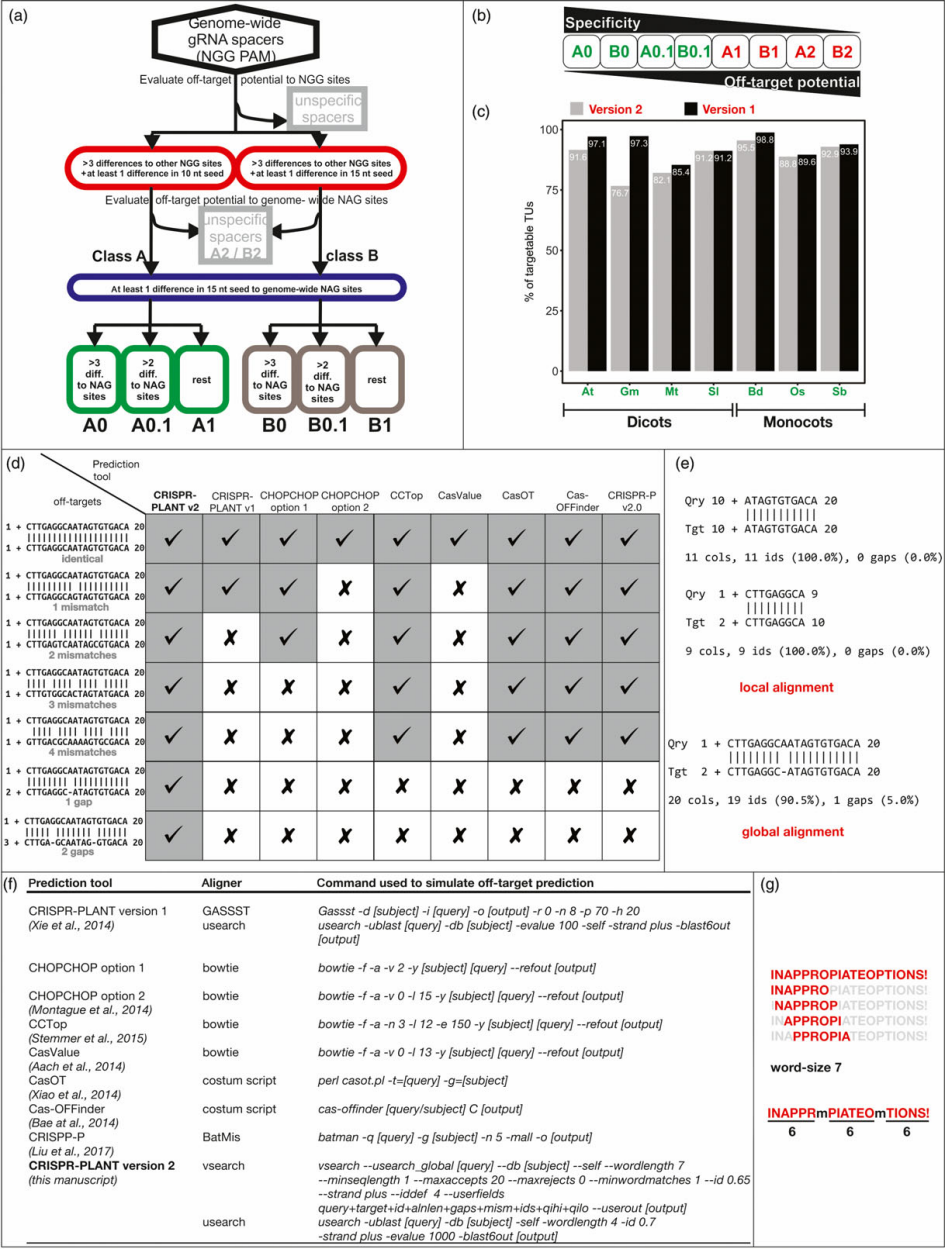
An improved off‐target analysis resulted in genome‐wide prediction of highly specific CRISPR/Cas9 spacer sequences for seven plant genomes. (a) Pipeline used to detect off‐targets and to classify spacer sequences. (b) Classes for spacers ordered by specificity and off‐target potential. (c) Comparison of highly specific targetable transcription units between the new CRISPR‐PLANT v2 and the old v1. (d) Seven different off‐target prediction tools were tested for their ability to find seven different potential off‐target sequences in an artificial rice chromosome 1 sequence. Checks indicate successful alignment and detection while crosses indicate a failure. (e) Examples of local and global alignment between a target sequence and an off‐target sequence with a one base pair deletion. (f) Exact programs, command and options used in the comparison of off‐target tools. (g) Example of words created by the word‐size seven, which is insufficient to detect sequences with two mismatches that are equally spaced out. At: *Arabidopsis thaliana*; Gm: *Glycine max*; Mt: *Medicago truncatula*; Sl: *Solanum lycopersicum*; Bd: *Brachypodium distachyon*; Os: *Oryza sativa*; Sb: *Sorghum bicolor*.

Based on the current reports that not all off‐targets could be predicted with current bioinformatics tools, we decided to test nine different tools for their ability to detect off‐targets (Figure 1d). We manually created seven off‐target sites with zero to four mismatches or one to two gaps. The mismatches or gaps in these sequences were equally spaced out because this type is the hardest to detect with inappropriate aligner options. We simulated the tools based on the aligner and options specified in the documentation to ensure that we could use our own test sequence for this evaluation (Figure 1f; Aach *et al*., 2014; Bae *et al*., 2014; Liu *et al*., 2017; Montague *et al*., 2014; Stemmer *et al*., 2015; Xiao *et al*.,^2014^). The results show that CRISPR‐PLANT v2 was able to detect every off‐target, but all other tools failed to detect a subset of the seven hidden sequences (Figure 1d). All other tools were also unable to detect our off‐target sequences with gaps. This is concerning because CRISPR/Cas9 possesses a considerable off‐target activity on sites with one to three mismatches or one gap, even if one or two of these differences are in the spacer seed region (Fu *et al*., 2013; Lin *et al*., 2014; Tsai *et al*., 2015). Most tools use Bowtie; however, Bowtie is designed to map a large set of up to 1024 bases long DNA when only one hit is expected in the genome (Langmead *et al*., 2009). Bowtie is not a general‐purpose aligner. Bowtie does not report gapped alignments and has a top‐strand bias when run in default mode (http://bowtie-bio.sourceforge.net/manual.shtml). Considering these limitations, Bowtie may be a fast but poor choice for predicting off‐target sites. Therefore, we decided to develop a new strategy that could circumvent most of these problems and lead to a higher sensitivity.

Aligners either use global or local alignment to compare sequences. If a sequence has an insertion or deletion when compared to the query, a local alignment may divide the string in two sub‐strings at the gap (Figure 1e). A global alignment will lead to only one alignment result including the full length of both strings. For CRISPR/Cas9 off‐target prediction, global alignments allow for a better detection of gapped off‐targets because the aligner result can directly inform about sequence similarity including the gap (Figure 1e). In addition, the options with which the program is evoked greatly affect the outcome. To speed up the search process, most aligners divide the strings into unique pieces of similar length called k‐mers, with k being the word‐size. The strings are then probed for common k‐mer occurrence, and a full alignment is only performed if a match is found (Figure 1g). The word‐size significantly limits detection of off‐targets because of the small size of spacer sequences. It is negatively correlated with the number of equally spaced out differences that can be detected. When choosing default range, a subset of off‐targets will be missed by the aligner. In order to detect all putative off‐targets, the word‐size should be five or smaller. We found that by carefully choosing aligners and their options, the sensitivity of CRISPR/Cas9 off‐target prediction can be significantly improved, which in return allows us to discover highly specific target sequences.

Here, we combined results from global and local alignments with optimized options of the genome‐wide NGG spacer sequences against genome‐wide NGG as well as NAG spacer sequences. Our new strategy yielded the highest sensitivity among all tested off‐target prediction tools (Figure 1d). It should be noted that this finding holds only true for the most challenging to detect sequences with equally spaced out differences. Even the worst performing off‐target prediction tool can detect an off‐target site as long as the mismatches still allow for a high enough common k‐mer. CRISPR‐PLANT v1 already used combined global and local alignments (Xie *et al*., 2014); however, options were not optimized for small sequences. Therefore, v1 also struggled to detect sequences with equally spaced out differences (Figure 1d). Since our v2 pipeline is expected to better predict possible off‐targets, it may eliminate more potentially specific spacers compared with v1. We would expect a lower number of targetable transcription units for the improved pipeline if this is the case. When comparing the improved strategy with the data from v1, we indeed found that the spacers in CRISPR‐PLANT v2 provide a smaller percentage of targetable transcription units (Figure 1c). This supports the assumption that the improved pipeline actually yielded an enhanced sensitivity and that v1 might have missed some off‐target sites that it was unable to detect because of inappropriate aligner options. We were able to expand prediction to these more challenging putative off‐targets, and CRISPR‐PLANT v2 provides a new and more specific list of spacer sequences for seven plant genomes. The selected species, namely *Arabidopsis thaliana*,* Brachypodium distachyon*,* Oryza sativa* (rice), *Medicago truncatula*,* Glycine max* (soybean), *Solanum lycopersicum* (tomato) and *Sorghum bicolor* are either an important model system or a crop species for food, feed, forage or biofuel.

For these species, all spacers provided by CRISPR‐PLANT v2 have sufficient specificity to other NGG sites in the genome (Figure 1a). But the NGG spacers were divided into group A with at least one difference in the 10 nt seed region, and B with least one difference in the 15 nt seed region in addition to three or more differences (Figure 1a). Different studies suggest slightly different lengths for the *Sp*Cas9 seed region, and the user can decide for a more conservative approach with a 10 nt seed for group A (Cong *et al*., 2013; Jinek *et al*., 2012; Tsai *et al*., 2015). In the last step, groups A and B were further divided by their potential to NAG off‐targets (Figure 1a). Classes A0, B0, A0.1 and B0.1 provide sufficient differences to other NGG and NAG targets, while classes A1, B1, A2 and B2 might have potential NAG off‐targets (Figure 1b). We recommend using A1 to B2 spacers only if no spacers from the high‐specificity classes A0 to B0.1 can be found for the target region of interest.

In this study, we described how current off‐target prediction tools were inaccurately developed, especially in regard to the choice of aligner and their options. We showed that we achieved an improved specificity by optimizing aligners and their options to better fit short sequences like the 20 nt spacer sequences used to guide Cas9. Based on these findings, we developed a new pipeline to perform a genome‐wide analysis of specific gRNAs for seven important model and crop plants. Our new strategy provided a slightly lower but still impressive number of highly specific spacer sequences that can be used to target 64.5%–92.6% of the coding sequences in the seven analysed plant genomes (Figure 1c). We assume these spacers are of highest quality compared to previously published gRNA spacer predictions because we were able to improve detection of rare but important off‐target sequences. Our results are available at http://www.genome.arizona.edu/crispr2/ and can be searched by either gene locus or region of a specific species. In addition, users can clone the analysis pipeline from https://github.com/bminkenberg/CRISPR-PLANTv2 to apply it to any genome or Cas variant of their choice.

## Author Contributions

B.M. designed the study, performed the bioinformatics analysis and wrote the manuscript. Y.Y. supervised the study and edited the manuscript. K.X. designed the original website and reviewed the manuscript. J.Z. updated the data on the website and administrated it. All authors reviewed and approved the manuscript.
